# A controlled trial of acute effects of human exposure to traffic particles on pulmonary oxidative stress and heart rate variability

**DOI:** 10.1186/s12989-014-0045-5

**Published:** 2014-11-01

**Authors:** Robert J Laumbach, Howard M Kipen, Susan Ko, Kathie Kelly-McNeil, Clarimel Cepeda, Ashley Pettit, Pamela Ohman-Strickland, Lin Zhang, Junfeng Zhang, Jicheng Gong, Manoj Veleeparambil, Andrew J Gow

**Affiliations:** Department of Environmental and Occupational Medicine and the Environmental and Occupational Health Sciences Institute, Rutgers Robert Wood Johnson Medical School, 170 Frelinghuysen Rd, Piscataway, NJ 08854 USA; Department of Molecular Genetics, Cleveland Clinic, 9500 Euclid Avenue, Cleveland, OH 44195 USA; Biostatistics, Rutgers School of Public Health, 675 Hoes Lane, Piscataway, NJ 08854 USA; Nicholas School of the Environment and Duke Global Health Institute, Duke University, 450 Research Dr, Durham, NC 27708 USA; Pharmacy and Toxicology, Rutgers University, 160 Frelinghuysen Road, Piscataway, NJ 08854 USA

**Keywords:** Particles, Traffic, Vehicle emissions, Oxidative stress, Heart rate variability, Exhaled breath condensate, Nitrite, Nitrate

## Abstract

**Background:**

For many individuals, daily commuting activities on roadways account for a substantial proportion of total exposure, as well as peak-level exposures, to traffic-related air pollutants (TRAPS) including ultrafine particles, but the health impacts of these exposures are not well-understood. We sought to determine if exposure to TRAPs particles during commuting causes acute oxidative stress in the respiratory tract or changes in heart rate variability (HRV), a measure of autonomic activity.

**Methods:**

We conducted a randomized, cross-over trial in which twenty-one young adults took two 1.5-hr rides in a passenger vehicle in morning rush-hour traffic. The subjects wore a powered-air-purifying respirator, and were blinded to high-efficiency particulate air (HEPA) filtration during one of the rides. At time points before and after the rides, we measured HRV and markers of oxidative stress in exhaled breath condensate (EBC) including nitrite, the sum of nitrite and nitrate, malondialdehyde, and 8-isoprostane. We used mixed linear models to evaluate the effect of exposure on EBC and HRV outcomes, adjusting for pre-exposure response levels. We used linear models to examine the effects of particle concentrations on EBC outcomes at post-exposure time points.

**Results:**

Mean EBC nitrite and the sum of nitrite and nitrate were increased from baseline at immediately post-exposure comparing unfiltered to filtered rides (2.11 μM vs 1.70 μM, p = 0.02 and 19.1 μM vs 10.0 μM, p = 0.02, respectively). Mean EBC malondialdehyde (MDA) concentrations were about 10% greater following the unfiltered vs. filtered exposures, although this result was not statistically significant. We found no significant associations between exposure to traffic particles and HRV outcomes at any of the time points. At immediately post-exposure, an interquartile range increase in particle number concentration was associated with statistically significant increases in nitrite (99.4%, 95% CI 32.1% to 166.7%) and nitrite + nitrate (75.7%, 95% CI 21.5% to 130.0%).

**Conclusions:**

Increases in markers of oxidative stress in EBC may represent early biological responses to widespread exposures to TRAPs particles that affect passengers in vehicles on heavily trafficked roadways.

## Background

Exposure to traffic-related air pollutants (TRAPs) has been associated with cardiovascular and respiratory health effects [1-3]. Exposure to TRAPs is highly prevalent, and for many individuals, a substantial proportion of daily exposure to TRAPs occurs during work commutes in rush-hour traffic [4]. Although the health effects of these short-term, intense exposures to TRAPs are not well-established, a case-crossover study found increased risk of myocardial infarction within hours of commuting activities [5]. Controlled exposure to emissions from diesel engines, a major source of TRAPs, caused acute respiratory irritation, inflammation, and adverse cardiovascular effects among human volunteers [6-12]. Short-term exposures to TRAPs on or near roadways have been associated with increased respiratory tract inflammation, decreased lung function, and changes in heart rate variability (HRV) [13-19].

Oxidative stress is a general mechanism by which exposure to TRAPs may cause adverse health effects [2,20]. Complex TRAPs mixtures include known primary oxidants, notably PAH-quinones and transition metals found in the particle phase [21-23]. Cellular interactions with TRAPs cause secondary production of oxidants, including nitric oxide and nitrosative compounds, as well as reactive oxygen species [24,25]. Oxidative stress has been implicated in inflammatory responses to diesel exhaust and diesel exhaust particles in cell systems, animal models, and the human respiratory tract [26,27].

Pulmonary oxidative stress can be sampled non-invasively with exhaled breath condensate (EBC). In EBC, concentrations of the nitrite and nitrate, relatively stable oxidation products of nitric oxide metabolism, have been associated with previous-day exposure to coarse particles among adults with pulmonary disease in European cities [28], and with levels of air pollution in healthy adults during the Beijing Olympics time period [29]. We found increased EBC nitrite and nitrate among adults with asthma after controlled exposure to diesel exhaust [9]. Increased concentrations of the lipid peroxidation products malondialdehyde (MDA) and 8-isoprostane have been associated with exposure to PM and traffic among children with asthma in Mexico City [30] and with ambient PM among students in Beijing [31,32].

Exposures to ambient and traffic-related particulate matter (PM) have been associated with adverse cardiovascular effects, including changes in heart rate variability (HRV), an indicator of the relative balance of parasympathetic and sympathetic autonomic control of heart rate that has predicted mortality in at-risk patient groups [33]. Polymorphisms in antioxidant genes modify associations between ambient PM and HRV, suggesting a link between oxidative stress and the autonomic effects of exposure to PM [34,35].

We hypothesized that 1.5-hr rides as a passenger in an automobile in morning rush-hour traffic on a major US highway causes acute increases in oxidative stress in the respiratory tract and changes in autonomic balance among healthy young adults, and that the PM component of the TRAP mixture is responsible for these effects. We measured changes in biomarkers of oxidative stress in EBC and HRV in a randomized, controlled, cross-over study design in which subjects wore a powered air purifying respirator (PAPR) during two car rides, and were blinded to High Efficiency Particulate Air (HEPA) filtration during one of the two car rides. Both inside the respirator and inside the vehicle cabin, we measured PM_2.5_ (particulate matter with median aerodynamic diameter cut-point of <2.5 μm) and particle number (PN) concentration as a proxy for ultrafine particles on roadways in traffic.

## Results

### Demographics

The subjects (mean age 22 years old) were predominantly male, reflecting the population of our academic campus at Rutgers University (Table 1).

**Table 1:** **Characteristics of subjects**

**Characteristic**	**Mean (range)**
Age (years)	22.4 (18–41)
BMI (kg/m^2^)	23.8 (19.50-28.66)
**Race/ethnicity**	**n (%)**
Asian	9 (43)
Black	3 (14)
Hispanic	1 (5)
White	8 (38)
**Sex**	**n (%)**
Men	15 (71)
Women	6 (29)

### Exposure measurements

**Figure 1:**
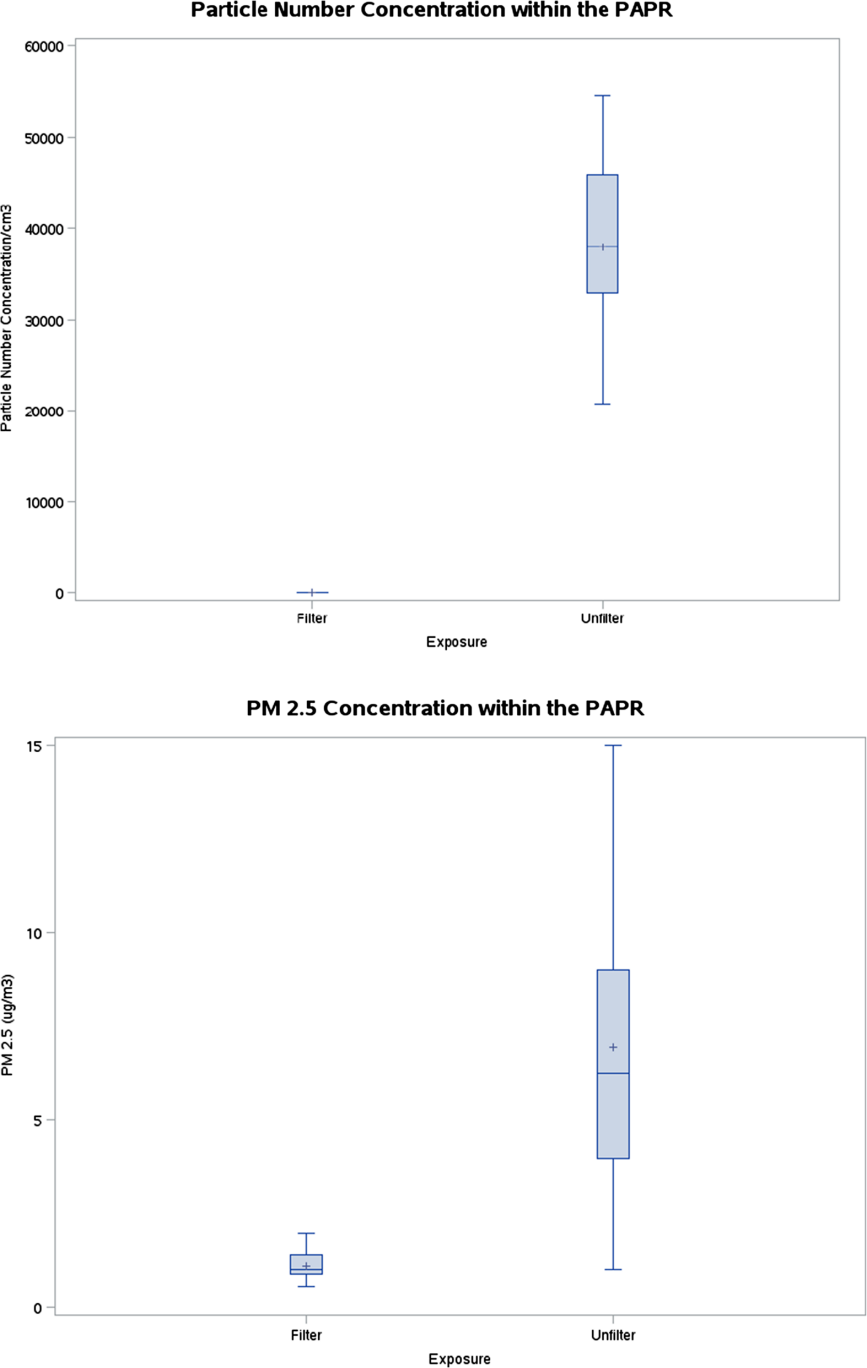
**Box plots showing mean, median, 25th percentile, 75th percentile, and range of concentrations of particle number and PM2.5 concentrations measured inside the respirator (PAPR) facepiece.**

With the HEPA filter in place, the mean (+/− SD) particle number concentration that subjects breathed inside the respirator hood was reduced by approximately 99.99% compared to unfiltered rides (3.5 +/− 4.1 vs. 37,999 +/− 9,545 pt/cm^3^) (Table 2) (Figure 1). The reduction in PM_2.5_ with HEPA filtration was of a much smaller magnitude (9.1 +/− 4.8 vs. 1.4 +/− 0.6 μg/m^3^). Black carbon and gas-phase pollutants were not measured inside the respirator. Mean concentrations of black carbon, CO, NO_2_, T and RH in the vehicle cabin air were similar on filtered vs. unfiltered days.

**Table 2:** **Concentrations (mean ± SD) of measured pollutants**

**Environmental measurements**	**Unfiltered rides**			**HEPA-filtered rides**		
	**Mean**	**SD**	**Median**	**Mean**	**SD**	**Median**
**Air inside vehicle cabin**						
Particle Number (cm^−3^)	41,350	12,678	40,806	44,411	16,115	40,063
PM_2.5_ (μg/m^3^)	11.7	6.3	10.7	12.2	6.2	10.3
BC (μg/m^3^)	6.1	3.6	5.5	6.2	2.5	6.2
CO (ppm)	1.1	0.4	1.1	1.3	0.4	1.2
NO2 (ppb)	26	9	26	26	11	24
Temp °C	24.5	2.9	24.2	23.6	2.4	23.8
RH%	25	10	28	27	10	28
**Air inside respirator facepiece**						
Particle number (cm^−3^)	37,999	9,545	37,928	3.5	4.1	2.2
PM2.5 (μg/m^3^)	9.1	4.8	8.2	1.4	0.6	1.3

*Abbreviations:* *HEPA* High-Efficiency Particulate Air.

Pollutant measurements were made in the vehicle cabin air and inside the facepiece of the respirator (particle number and PM2.5 only) during all rides under both unfiltered and filtered conditions.

Subjects wore the PAPR during both car rides, blinded to the presence or absence of the HEPA filter. The filter is not visible inside the PAPR, and the respirator is very quiet and maintains constant airflow and a positive pressure inside the loose-fitting face piece with or without the filter in place. Stress Questionnaire data showed no difference in perceived stress or anxiety during the car rides while wearing the PAPR compared to prior to the car rides while not wearing the PAPR [Stress Symptom Rating (SSR) mean score (SD): 2.05 (0.81) vs. 2.06 (0.95)].

### EBC markers

**Figure 2:**
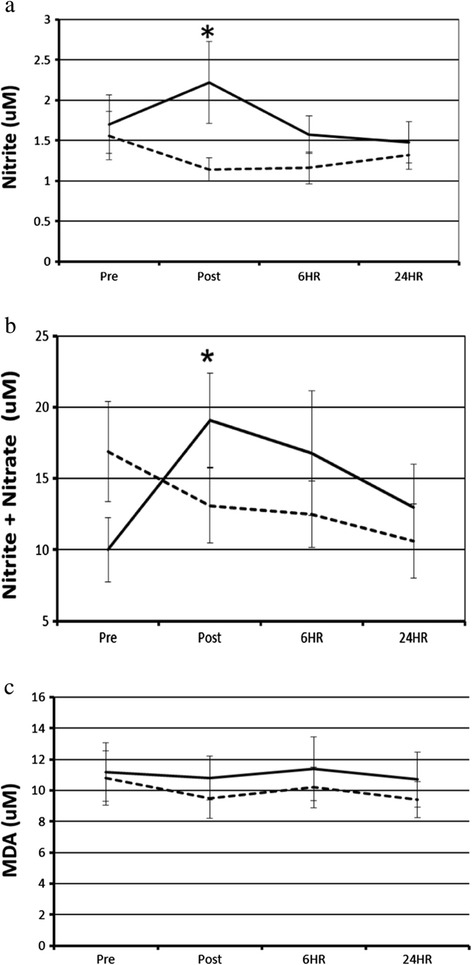
**EBC outcome measurements before and after breathing either HEPA-filtered or unfiltered air during car rides.** EBC nitrite **(a)**, nitrite + nitrate **(b)**, and MDA **(c)** at pre-exposure, immediately post-exposure, and 6 and 24 hr post-exposure with HEPA filtered breathing air (dashed line) or unfiltered breathing air (solid line). N = 20 for most data points, with missing single data points at some 6 and 24 hours (N = 19) *indicates significance at p = 0.02 for exposure effects comparing change from baseline for unfiltered rides to change from baseline for filtered rides.

Figure 2 shows concentrations of nitrite, the sum of nitrite and nitrate (nitrite + nitrate), and MDA in EBC collected at pre-exposure, post-exposure, 6 hr, and 24 hr after the car rides. Following the unfiltered rides, mean EBC nitrite was increased at post-exposure compared to baseline (2.11 μM vs 1.70 μM), whereas following the filtered rides, mean EBC nitrite decreased at post-exposure compared to baseline (1.14 μM vs. 1.56 μM) (p = 0.02 comparing change from baseline in filtered vs. unfiltered).

In the unfiltered rides, mean EBC nitrite + nitrate was increased at post-exposure compared to baseline (19.1 μM vs 10.0 μM), whereas in the filtered rides, mean EBC nitrite + nitrate decreased at post-exposure compared to baseline (13.1 μM vs 16.9 μM) (p = 0.02 comparing change from baseline in filtered vs. unfiltered). Changes in mean EBC nitrite + nitrate from baseline, comparing filtered and unfiltered rides, were not statistically significantly different at 6 hr and 24 hr after the rides.

Mean MDA concentrations in EBC were about 10% greater in the unfiltered vs. filtered conditions at post-exposure, 6 hr, and 24 hr, but these differences were not statistically significant. EBC 8-isoprostane was not detectable in a majority of the samples using our HPLC method.

In regression analysis of data from filtered and unfiltered rides, an interquartile range (IQR) increase in particle number concentration (36,392/cm^3^), measured inside the respirator face piece, was associated with statistically significant increases in nitrite and nitrite + nitrate at the immediately post exposure time point, but not at the 6 and 24 hr time points (Table 3). There were no statistically significant associations between PM_2.5_ (IQR 5.25 μg/m^3^ measured inside the respirator and EBC nitrite, nitrite + nitrate, or MDA.

**Table 3:** **Estimated% difference (95% CI) in EBC nitrite, nitrite + nitrate, and malondialdehyde (MDA) associated with interquartile range change relative to the median, adjusted for pre-exposure level, in particle number concentration (cm** ^**−1**^ **,PN) and PM2.5 inside the respirator for all car rides**

**EBC biomarker**	Pollutant**	**Time pt.**	**% Response/IQR increase**	
Nitrite	PN	0 hr	99.4%	(32.1% to 166.7%)*
		6 hr	22.3%	(−21.9% to 66.5%)
		24 hr	7.1%	(−16.2% to 29.8%)
	PM2.5	0 hr	27.6%	(−27.3% to 82.4%)
		6 hr	13.2%	(−18.6% to 45.0%)
		24 hr	−1.9%	(−10.0% to 6.2%)
Nitrite + Nitrate	PN	0 hr	75.7%	(21.5% to 130.0%)*
		6 hr	62.9%	(−35.5% to 160.3%)
		24 hr	33.5%	(−18.6% to 85.2%)
	PM2.5	0 hr	26.4%	(−13.7% to 66.5%)
		6 hr	8.9%	(−61.0% to 78.8%)
		24 hr	7.8%	(−33.0% to 48.6%)
MDA	PN	0 hr	11.3%	(−16.2% to 37.3%)
		6 hr	0.3%	(−43.2% to 42.7%)
		24 hr	15.1%	(−16.8% to 47.1%)
	PM2.5	0 hr	−1.4%	(−20.6% to 17.9%)
		6 hr	3.8%	(−27.0% to 34.5%)
		24 hr	1.1%	(−24.3% to 26.6%)

*p < 0.01 for change from baseline.

**Inter-quartile ranges: PN = 36,392, PM2.5 = 5.25, BC = 4.66.

### Heart rate and HRV

HRV parameters generally decreased from pre-exposure to 6 hr and 24 hr for both filtered and unfiltered rides, but there were no statistically significant differences between unfiltered and filtered rides (Table 4). Similarly, LF and total power increased from baseline to post-exposure, but with no statistically significant differences between unfiltered and filtered rides. There were no consistent trends or statistically significant effects of exposure (filtered vs. unfiltered) on HRV or HR at any time points. There was a large amount of variation in HRV outcome measurements both within and between subjects.

**Table 4:** **Heart rate variability (HRV) at time points before and after unfiltered and filtered care rides**

**Out-come**	**Exposure**	**Mean (SD)**				**Mean of differences from pre-exposure (SD)**		
		**Pre-exposure** ^**a**^	**Immediately Post** ^**b**^	**6 hours post** ^**a**^	**24 hours post** ^**c**^	**Immediately Post**	**6 hours post**	**24 hours post**
RMSSD (ms)	Filtered	0.095 (0.066)	0.103 (0.064)	0.064 (0.042)	0.082 (0.056)	0.008 (0.028)	−0.031 (0.043)	−0.011 (0.043)
	Unfiltered	0.073 (0.048)	0.091 (0.051)	0.056 (0.033)	0.077 (0.059)	0.017 (0.050)	−0.016 (0.046)	0.077 (0.070)
	*P-value assessing exposure effect* ^d^					*.5180*	*.91056*	*.9756*
LF (ms^2^)	Filtered	1152.6 (1549.4)	1575.8 (1447.1)	623.1 (511.1)	933.5 (1045.4)	423.2 (1339.5)	−529.6 (1263.5)	−237.6 (1573.3)
	Unfiltered	1112.2 (1005.2)	1432.9 (996.7)	752.9 (681.2)	827.9 (795.5)	281.3 (921.9)	−359.3 (733.0)	−284.0 (663.5)
	*P-value assessing exposure effect* ^d^					*.3750*	*.2530*	*.7847*
HF (ms^2^)	Filtered	2294.3 (3646.1)	2453.7 (2993.9)	1029.9 (1116.5)	1675.6 (2562.2)	159.4 (1232.4)	−1264.4 (3220.3)	−540.3 (2110.6)
	Unfiltered	1908.1 (2922.2)	1882.6 (1996.0)	787.4 (768.2)	1613.0 (3191.2)	−94.3 (1853.6)	−1120.7 (2560.8)	−222.9 (1456.3)
	*P-value assessing exposure effect* ^d^					*.2893*	*.2761*	*.6482*
LF/HF (ms^2^)	Filtered	1.2 (1.6)	1.1 (0.9)	1.4 (1.2)	1.0 (0.6)	−0.1 (1.1)	0.3 (1.7)	−0.2 (1.6)
	UnFiltered	1.2 (0.9)	1.3 (1.1)	1.3 (0.8)	1.1 (1.1)	0.1 (0.9)	0.1 (0.7)	−0.3 (0.7)
	*P-value assessing exposure effect* ^d^					*.3263*	*.5061*	*.8774*
Total Power (ms^2^)	Filtered	4644.2 (4699.9)	7879.1 (8187.1)	2699.3 (1975.1)	3891.0 (3658.0)	3234.9 (7109.1)	−1944.9 (4061.6)	−699.5 (3503.1)
	Unfiltered	4391.9 (4976.9)	6338.7 (5207.0)	2948.1 (2513.4)	3652.7 (4980.4)	1828.7 (3700.2)	−1443.8 (3190.1)	−670.1 (3559.9)
	*P-value assessing exposure effect* ^d^					*.2795*	*.4105*	*.9899*
HR (min^−1^)	Filtered	60.1 (9.5)	56.8 (7.9)	68.9 (12.8)	61.3 (8.2)	−3.3 (5.0)	8.8 (8.5)	0.3 (6.8)
	Unfiltered	61.4 (10.2)	59.4 (9.7)	70.2 (11.8)	63.3 (11.8)	−2.7 (7.9)	8.9 (9.3)	1.6 (6.8)
	*P-value assessing exposure effect* ^d^					*.4087*	*.6640*	*.5514*

^a^N = 20 for filtered; N = 21 for unfiltered; ^b^N = 20 for filtered and unfiltered; ^c^N = 19 for filtered N = 21 for unfiltered; ^d^Derived from mixed-effects linear model, controlling for baseline as a covariate and nested sessions within subject.

Means and Standard Deviations of HRV outcomes at each time point, the changes from pre-exposure and p-values assessing the effect of exposure (unfiltered vs. HEPA-filtered ride) on change in HRV outcomes from pre-exposure baseline to post-exposure time points.

## Discussion

Among young adults riding as passengers for 90–110 min in heavy motor-vehicle traffic, we found increased nitrite and nitrite + nitrate in EBC immediately after rides during which the subjects breathed unfiltered vehicle cabin air compared to rides during which they breathed HEPA-filtered air. In analysis of single pollutant effects, mean particle number concentration measured inside the respirator was associated with increased nitrite and nitrite + nitrate in EBC immediately after the car rides. MDA concentrations in EBC were ~12 to 14% greater at post-exposure and 6 and 24 hr after unfiltered compared to filtered rides, but these differences were not statistically-significant. We did not find statistically significant differences in HRV between unfiltered and filtered rides. These results suggest that exposure to the particle phase of TRAP contributes to acute, transient increases in respiratory tract oxidative stress among healthy adult vehicle occupants during short-term exposures to TRAP that occur during commuting.

We used an experimental approach with a filtered-air control condition in order to isolate the effects of exposure to TRAP particles on biomarkers of acute oxidative stress and autonomic balance that may mediate respiratory and cardiovascular health effects of relatively intense, short-term exposures to TRAPs. TRAPs are a complex mixture of primary gas-, vapor-, and particle-phase pollutants from engine emissions, as well as re-suspended road dust and products of vehicle and road wear. In various settings in close proximity to sources, such as while occupying a vehicle in traffic or residing near a major roadway, exposures to TRAP are likely to co-vary with noise, other environmental factors, and psychological stress. Observational epidemiologic approaches may have limited ability to disentangle the effects of specific components of TRAPs from the effects of these potentially confounding or effect-modifying co-exposures. Controlled human exposure studies in the laboratory can control for potential confounders, but generally with some sacrifice of relevance to complex real-world exposures. To control for these factors in a real-world setting, we randomized exposure to HEPA-filtered and unfiltered vehicle cabin air in a blinded, cross-over study design. The observed differences between unfiltered (particles and gas-phase TRAPs) and particle-filtered (gas-phase TRAPs only) exposures suggest that particles are necessary, although they may not be sufficient, to cause the observed increases in markers of oxidative stress in the respiratory tract after exposure to TRAPs.

This is the first report of acute increases in markers of oxidative stress in EBC following short-term, real-world exposure to TRAPs. In a recent controlled exposure study, we found increases in EBC nitrite in subjects with asthma immediately after a 1-hr exposure to diluted diesel exhaust compared to filtered-air control [9]. Balint et al. (2001) reported an immediate, transient increase in EBC nitrite + nitrate among smokers after smoking two cigarettes [36]. Other studies have found that EBC nitrite and/or nitrite + nitrate were positively associated with levels of ambient air pollution [28,29], as well as with the disease states of asthma, cystic fibrosis, and COPD [37-39].

We found statistically significant associations between particle number concentrations and EBC nitrite and nitrite + nitrate immediately following the car rides, but no associations between PM_2.5_ and these outcomes. Particle number is a more specific indicator of TRAP particles, which are predominantly in the ultrafine size range near emission sources on highways, compared to PM_2.5_ mass concentration, which includes generally larger ambient particles and is less influenced by TRAP particles [40]. Therefore, these results are consistent with the interpretation that exposure to TRAP particles, not ambient PM, were a primary cause of the airway response measured in EBC comparing unfiltered to filtered rides.

Increases in nitrite and nitrate in EBC may reflect increased NO production and/or increased oxidation of NO to nitrite and/or nitrite to nitrate. Although both EBC nitrite and fractional exhaled nitric oxide (FENO) can indicate the level of NO production within the lung, there are significant differences between the two measures. By definition, FENO is a measure of NO released to the gas phase that has escaped reaction with aqueous components of the lung lining. In contrast, nitrite represents NO that has been “fixed” in the aqueous phase by oxidative reactivity. Therefore, EBC nitrite is a more complex measure as it is determined by the rates of NO production and oxidation. The immediate increase in EBC nitrite was consistent with increased NO production from the constitutive isoforms of nitric oxide synthase (NOS), rather than inducible NOS2 activity, which requires protein synthesis. Consistent with increased production of NO in response to TRAP exposure, Adar et al. found that exposures to PM_2.5_ and black carbon were positively associated with FENO among elderly passengers on group diesel bus rides in St. Louis [41]. Even if the mechanism of response to inhaled TRAP particles were solely increased NO production, this represents a source of oxidative stress through NO’s role as an oxidant.

The observed increase in MDA, a marker of lipid peroxidation, after unfiltered rides compared to filtered rides (Figure 1c) was consistent across time points, but not statistically significant. Longer-term exposures to air pollutants have been associated with MDA in EBC [30,31]. Among a panel of healthy adults, higher levels of EBC MDA were measured during the pre- and post-Olympic periods, compared to the Olympic period in Beijing, China when air pollutants were drastically reduced [31].

Comparing filtered to unfiltered rides, we found no statistically significant changes from baseline in any measured HRV parameters at post-exposure, 6, or 24 hr (Table 3). A recent meta-analysis of studies that included exposure to ambient or occupational PM demonstrated a significant inverse relationships between PM_2.5_ and LF, HF, RMSSD and SDNN HRV measurements [33]. Fewer studies have examined the effects of direct exposure to TRAPs on HRV, with inconsistent results [14,19,42,43]. The acute effects of diesel exhaust on HRV have been inconsistent in controlled exposure studies [44,45].

In a similar study design, Langrish et al. (2009) found that 24-hr SDNN and LF HRV were higher after 2-hr walks in central Beijing during which healthy subjects wore a negative pressure, air purifying (“N95”) respirator, compared to walks without the respirator [12]. Negative pressure, air-purifying respirators are known to affect cardiovascular physiology including HR and BP, and subjects could not be blinded to the exposure conditions [46,47]. In the present study, we used a powered air purifying respirator that does not alter pulmonary mechanics, and subjects were blinded to the presence or absence of a HEPA filter in the light-weight, hood-type, respirator, which was worn for both filtered and unfiltered rides.

### Limitations

The study was small and may have been underpowered to detect some effects that may be of biological significance, particularly changes in HRV and EBC MDA, especially in light of the relatively low exposure levels. The young healthy, university student subjects do not represent the general population and may not reflect the response of more susceptible populations. The comparisons between rides were between exposures to combined particle- and gas-phases (unfiltered rides) versus gas-phase TRAPs only (filtered rides). Therefore, we cannot attribute the observed effects on nitrite and nitrite + nitrate in EBC to the particles alone. It is possible that an interaction between particles and gas-phase compounds is essential to produce these effects.

We could not control the levels of exposure to PM during unfiltered rides and the variability in mean particle number concentration and PM_2.5_ measured inside the vehicle cabin were large (IQR 18,080 particle/cm^3^ and 6.2 μg/m^3^, respectively). The relatively low levels of PM in the vehicle reflected real-world exposure conditions, but limited our power to see effects. Traffic pollutant mixtures in vehicles vary in concentration and in composition with different ambient and near-roadway conditions, vehicle types, and operating conditions [4].

The ~99.99% reduction in particle number concentration with HEPA filtration (Table 2) is consistent with the expected, highly-efficient removal of ultrafine traffic particles. We speculate that the smaller reduction in PM_2.5_ of about 85% was due to re-suspension of previously-deposited larger particles from the subjects’ hair and skin surfaces as filtered air was delivered above the subject’s head before flowing down to the subject’s immediate breathing zone where we positioned the sampling ports. We believe these particles entered the breathing zone air downstream of the HEPA filter, which is rated at ≥99.97% removal of 0.3 μm particles and is expected to be even more efficient at removing the larger particles that contribute to PM _2.5_ than the smaller particles that dominate the particle number concentration on roadways. However, we have not characterized these particles.

Several investigators have questioned the usefulness of EBC nitrite as a biomarker reflective of the lower respiratory tract [48,49]. A study of subjects with and without tracheostomy suggested that chemical reduction of salivary nitrate to nitrite by oropharyngeal bacteria made a substantial contribution to nitrite concentrations in EBC that was collected by oral breathing [48]. Dietary nitrate was found to strongly influence salivary nitrate and EBC nitrite concentrations. In our study, subjects fasted prior to the baseline and post-exposure EBC collections, and then were asked to eat similar foods during the post-fast meals after filtered and unfiltered rides. Control of food consumption, along with the within-subjects, repeated-measures design, may have mitigated any effect of oropharyngeal contamination, which would likely be nondifferential, tending to bias results towards the null hypothesis.

## Conclusions

In this study, nitrite and nitrite + nitrate in EBC were increased immediately after 1.5 hr car rides when subjects breathed unfiltered vehicle cabin air, but not after rides when subjects breathed HEPA-filtered air, suggesting that TRAP particles play an essential role in these effects. There were no statistically significant differences in HRV or HR after unfiltered compared to filtered rides. The health significance of the observed effects is not known, but they may be indicative of early biological responses to widespread exposures to TRAPs among passengers in vehicles on heavily trafficked roadways.

## Methods

We recruited 21 nonsmoking, healthy adults without serious chronic illness, pulmonary or cardiovascular disease, atrial flutter, atrial fibrillation, or cardiac pacing. All subjects lived on campus or within 10 miles of the clinical center Clinical Center of the Environmental and Occupational Health Sciences Institute (EOHSI) in Piscataway, NJ, except for one subject who lived 15 miles away. Eight subjects rode campus diesel buses from residences on the university campus; the remainder drove personal vehicles. The study protocol was approved by the University of Medicine and Dentistry of New Jersey Institutional Review Board.

### Study procedure

**Figure 3:**
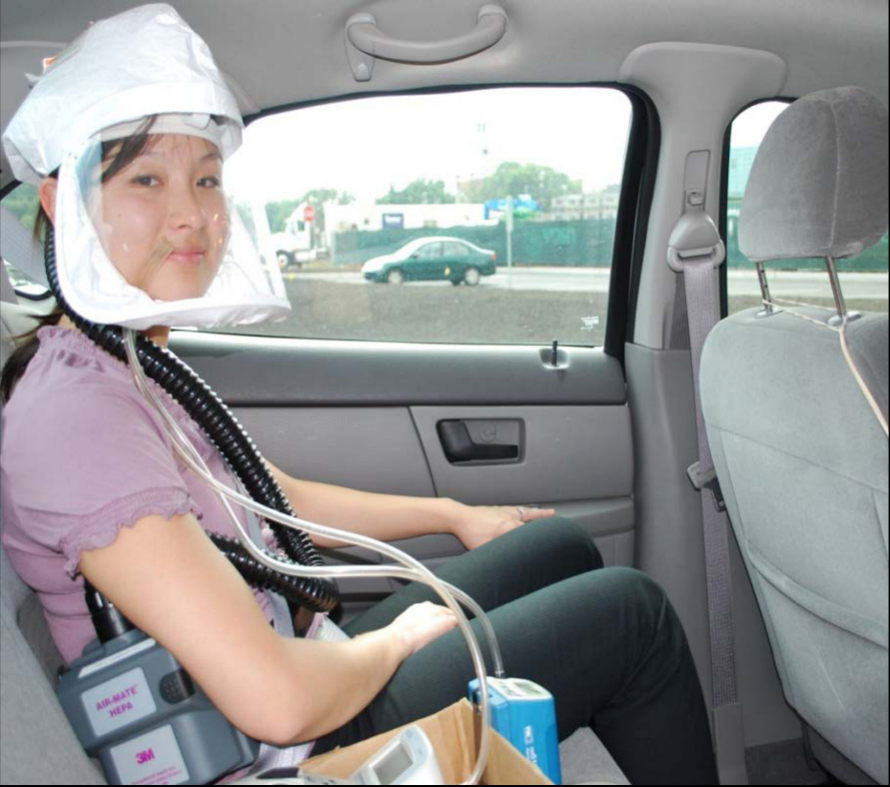
**An investigator demonstrates set-up of the powered air purifying respirator, with sampling instrument inlets in place inside the respirator facepiece, as worn by subjects.**

Each subject participated in two nominally 1.5 hr (range 90–110 min, depending on traffic) car rides in morning Monday-Friday rush-hour traffic, at least one week and no more than six weeks apart, in a 2002 Ford Taurus sedan. Subjects wore a powered air-purifying respirator (PAPR, Airmate™, 3 M, Minneapolis, MN) with a HEPA filter present during one ride (filtered ride), and absent during the other ride (unfiltered ride), in randomized order (Figure 3). Subjects and technicians were blinded to the presence or absence of the filter. Outcomes were measured before (pre-exposure), after (post-exposure), and at 6 and 24 hrs after the car rides began. To avoid effects of food ingestion on study outcomes, subjects fasted after midnight. Subjects were instructed to avoid major roadways if driving to the Clinical Center. Subjects reported at 7:00 AM for the pre-exposure session, which included a current stress symptom rating (SSR), consisting of five-point modified-Likert scales between two pairs of antonyms for “stress” (tense-relaxed; stressed-at ease) and “anxiety” (nervous-calm; jittery-tranquil). After electrocardiogram (ECG) recordings and EBC collection, subjects underwent non-invasive studies of endothelial function, and a peripheral venous blood draw, results of which will be reported elsewhere.

The subject was seated in the rear seat of the vehicle, which was driven by a professional driver from the EOHSI Clinical Center to and from the northernmost point on the New Jersey Turnpike (NJTPK; 79 miles roundtrip, of which 55 miles were on the NJTPK, 18 miles on Interstate 287, and 6 miles on local secondary roads). The NJTPK is a highway with six traffic lanes in each direction. The study vehicle remained in the right-most lane, designated for both trucks and cars, averaging 65 mph when unimpeded by traffic. Stop-and-go traffic conditions were rarely encountered, and 50% of the rides lasted between 95 and 100 minutes. The vehicle ventilation settings were maintained with the vent “open” and fan at the medium setting. Other climate control settings were adjusted for subject comfort. Midway through the car ride, the subject completed another SSR questionnaire. The subject returned to the EOHSI clinic immediately (post-exposure) and at 6 hr and 24 hr after the car ride for outcome measurements. Subjects ate a meal consisting of the same foods between the post-exposure and 6 hr time points. For the 24-hr time-point, the subjects fasted again after midnight and returned to the EOHSI clinic at 7:00 AM to repeat a protocol identical to the pre-exposure protocol.

### Exposure measurements

We made the following measurements with inlets at the subject’s breathing zone inside the respirator face piece and near the center of the vehicle cabin: mass concentrations of PM with median cut-point of 2.5 μm (PM_2.5_) at 1-min intervals using TSI SidePak model AM510 aerosol monitors (TSI, Inc, Shoreview, MN) with the calibration factor set at 0.32 (based on collocated gravimetric analysis of local ambient); number concentrations of particles with aerodynamic diameter from 0.01 to 1.0 μm at 1-min intervals using a condensation particle counter, TSI model 3007 (TSI, Inc.). Measured in the vehicle cabin only: back carbon using an AE-51 microaethalometer (Aethlabs, San Francisco, CA) at flow rate of 100 ml/min and 1-min time base; nitrogen dioxide (NO_2_) collected on triethanolamine-coated Sep-Pak cartridges (Waters, Corp, Millford, MA), analyzed using HPLC-UV as previously reported [13]; carbon monoxide (CO) and air temperature were measured continuously with a Langan T15v monitor (Langan, Inc, San Francisco, CA); and humidity with a HOBO 8 Pro Series monitor (Onset, Bourne, MA).

### Exhaled breath condensate

We collected 1–2 ml of EBC using an EcoScreen device (Jaeger, Wurzburg, Germany) with 20 minutes of tidal breathing. We triple rinsed all surfaces with nitrite-free water prior to contacting the EBC, and froze samples at −80 C for later analysis. We measured concentrations of nitrite and nitrite + nitrate using selective catalytic reduction and chemiluminescence detection (NOA 280i, GE Analytics, Boulder Co.). The detection limits and precision for nitrite and nitrite + nitrate were 1 μM and 8.2% (measured as %RSD from 10 replicates) and 2.5 μM and 12.0% (6 replicates), respectively.

For MDA analysis, a mixture of EBC, phosphoric acid, and thiobarbituric acid, was heated and injected into an HPLC-fluorescence system [50]. The detection limit, recovery and precision of this method were 1.8 nM, 75.9%, and 2.2% (8 replicates), respectively.

Concentrations of 8-isoprostane in EBC samples were analyzed using a modified immunoaffinity purification combined with a LC-MS/MS method [51]. After addition of 0.25 ng of 8-iso-PGF_2α_-D_4,_ the extract from 8-isoprostane affinity sorbent (Cayman, Ann Arbor, MI, USA) was analyzed on a quadrupole mass spectrometer (ThermoFisher Scientific, San Jose, CA, USA). The method recovery was 98.4% with analytic precision (8 replicates) of 11.2% and a detection limit of 2.5 pg/mL.

### Heat rate variability

A two-channel, three-lead, Holter monitor (Trillium 3000; Forest Medical, East Syracuse, NY) recorded ECG for 12 min with the subject lying supine at the pre-exposure, post-exposure, 6-hr and 24-hr time-points. For HRV analysis, the best quality (minimum artifact by visual inspection), continuous 5-min period was selected from the last 7 min of the 12-min recording. We processed the digital ECG signal, sampled at 256 Hz, and calculated the HRV parameters using PC-based software (Trillium Gold; Forest Medical, East Syracuse, NY), after reviewing and correcting mislabeled beats or artifacts. We included all normal-to-normal (NN) intervals in the 5-min recording in computations of the standard deviation of normal to normal intervals (SDNN), square root of the mean of the squared differences between adjacent NN intervals (r-MSSD), high-frequency power (HF; 0.15 to 0.4 Hz), low-frequency power (LF; 0.04 to 0.15 Hz), and LF:HF ratio.

### Statistical analysis

We used mixed linear models to evaluate the effect of exposure (unfiltered vs. filtered) on EBC and HRV outcomes, with post-exposure measures as the response, exposure as the predictor, and pre-exposure level of the corresponding measure as a covariate, with a random effect for subject. We used linear models to examine the effects of single pollutant levels during unfiltered rides on EBC markers at post-exposure time points. Statistical significance was set at α = 0.05.
